# Evaluating the use of drones within medical logistics: A patient-focused qualitative evaluation

**DOI:** 10.1371/journal.pdig.0001459

**Published:** 2026-06-25

**Authors:** Colin B. Shore, Ashley Shepherd, Frances Hines, Hazel Dempsey, Karen Bell, Dylan Powell

**Affiliations:** 1 School of Health and Life Sciences, Glasgow Caledonian University, Glasgow, United Kingdom; 2 Faculty of Health Sciences and Sport, University of Stirling, Stirling, United Kingdom; 3 NHS Highland RD&I Division, Inverness, United Kingdom; 4 NHS Grampian Innovation Hub, PIP Directorate, NHS Grampian, Aberdeen United Kingdom; Iran University of Medical Sciences, IRAN, ISLAMIC REPUBLIC OF

## Abstract

Drone technologies offer significant promise for enhancing medical logistics, particularly in rural and remote areas. However, despite several pilot trials across the UK, there remains a lack of evidence regarding patient perceptions of drone integration in routine healthcare delivery. This study aimed to explore patient perspectives on the use of drones in medical logistics, with a focus on cost, safety, and impact on quality of life amongst three medical areas (chemotherapy, lab diagnostics and blood products). A qualitative study using semi-structured interviews and small focus groups was conducted with 26 participants from five Scottish health boards. Participants were recruited via NHS engagement leads, community networks, and snowball sampling. Data were collected via Microsoft Teams and analysed thematically using an inductive approach. Eight key themes emerged: Acceptance, Knowledge of drones, Potential use within the NHS, Potential impact, Concerns, Public perception, Support networks, and Future innovation. Participants expressed cautious optimism, highlighting potential benefits including improved access in rural areas, reduced travel burdens, and environmental gains. Concerns included technical reliability, regulatory complexity, data privacy, and the risk of increasing health inequities. Drones were viewed by patients as a promising complement to existing medical logistics, especially for emergency and rural applications. However, successful integration demands transparent communication, co-design with stakeholders, robust regulatory planning, and sustainable funding models to ensure equitable and safe implementation.

## Author summary

We explored how patients feel about the use of drones to transport medical supplies and samples within the National Health Service. Although drones have shown technical promise in improving delivery speed and reliability, their successful use in healthcare depends on whether patients understand and trust this technology. We spoke with people across several Scottish health boards to learn about their expectations, hopes, and concerns. Most participants saw drones as a practical solution to the challenges of delivering care in rural and remote areas, with benefits such as faster access to treatment, lower travel costs, and reduced environmental impact. At the same time, they raised questions about safety, privacy, and fairness between communities. By focusing on patients’ experiences and perspectives, our research helps ensure that future drone services are introduced transparently and designed around the needs of those they aim to serve.

## Introduction

Healthcare systems worldwide face increasing pressure due to rising demand, workforce shortages, and demographic challenges such as ageing populations and multimorbidity. Scotland, similar to many nations, is experiencing escalating healthcare demand coupled with persistent shortages of skilled healthcare professionals, a situation further exacerbated by recent global disruptions including the COVID-19 pandemic, geopolitical instability, and significant inflationary pressures. Current projections indicate that continuing with existing healthcare models will necessitate approximately 84 million additional health workers globally by 2030 with a projected shortfall of 18 million, underscoring the urgent need to explore innovative strategies and technologies to enhance healthcare delivery. In response to these mounting pressures, healthcare innovation-particularly through the integration of new technologies-has become increasingly critical. Many industries outside healthcare have rapidly embraced technological advancements, demonstrating substantial gains in efficiency and service delivery. Healthcare, albeit traditionally slower relative to other industries in the widespread adoption of new technologies, has made significant progress in recent years, propelled by the necessity to address workforce limitations and improve patient outcomes through innovative practices [1,2].

Rural healthcare delivery poses unique logistical challenges, which are especially pronounced in Scotland, where rural areas constitute 98% of the land mass but only host 17% of the population [3]. Traditional methods of medical logistics in these areas often lead to delays and inefficiencies. Recognising these challenges, the Argyll and Bute Health and Social Care Partnership trialled drone technology in 2021 to transport COVID-19 test kits and results between the Scottish mainland and the Isle of Mull. This trial demonstrated considerable promise, reducing a journey traditionally taking up to 36 hours by road and ferry to just 15 minutes via drone, highlighting drones’ potential in revolutionising medical logistics in rural and isolated regions [4,5].

Despite clear operational advantages in terms of speed, efficiency, economic viability, and environmental sustainability and a broad understanding of the publics’ perception of drones across sectors significant gaps remain in understanding patient perspectives toward drone integration in healthcare logistics [6]. Prior research into medical drone applications has predominantly focused on technical feasibility, operational efficiencies, and environmental impacts, with limited exploration of patient views and the wider implications for patient quality of life. Preliminary advisory consultations conducted prior to this study identified several critical factors from the patient perspective, including safety concerns, convenience, cost-effectiveness, scalability, efficiency, traceability, and authenticity [6–9]. To address these gaps, the current study aimed explicitly to explore and understand patient perceptions regarding three key dimensions of drone use in medical logistics: (1) cost and efficiency, (2) safety and reliability, and (3) the potential impacts on patient quality of life. This study specifically focused on three high-impact medical areas: chemotherapy, laboratory diagnostics, and blood products. Investigating these patient perspectives is essential for ensuring that the adoption of drone technology aligns with patient expectations and needs, ultimately facilitating more patient-centred and effective healthcare innovation.

## Materials and methods

### Study design

A qualitative methodology was employed to explore patient perspectives on the integration of drones into medical logistics. Ethical approval was granted by the University of Stirling NHS, Invasive or Clinical Research Committee (Reference: NICR 2024 17743 13326). In support, following ethical approval, letters of no objection were obtained from the research and development (R&D) teams at NHS Highland, NHS Ayrshire and Arran, and NHS Grampian.

### Participant recruitment

Health boards [10] were approached on the basis of ensuring a diverse representation from contrasting regions. Due to the concept of drone use, focus was placed on health boards enabling the exploration of perspectives specific to rural and remote areas. Participants were recruited through a purposive sampling approach to ensure a diverse cohort representing a range of ages, genders, backgrounds, and medical experiences, including both physical and cognitive health conditions. Eligible participants included those with healthcare experience in the delivery of medical supplies and medications, specifically related to chemotherapy, laboratory diagnostics, and blood products. Recruitment was facilitated through opportunity based and snowball recruitment strategies to identify participants meeting the criteria.Engagement leads within relevant NHS research and governance teams, who distributed study invitations and posters via email to appropriate individuals and groups. For participants identified through support networks (e.g., prostate cancer support groups), direct contact details for the University of Stirling research team were provided. In instances where local charities or community groups were approached, a study advertisement was disseminated via email newsletters or social media platforms such as Facebook, encouraging interested individuals to register their interest directly with the research team. Snowball sampling was also employed to widen participant reach. Participants who registered their interest were sent an information sheet and an outline of the interview process before scheduling a convenient session time. A £25 e-voucher was provided to participants as a token of appreciation for their involvement, as detailed in the Ethics application. A purposive sample size with an upper limit of 30 participants was selected. It was felt that this would provide sufficient depth and richness of data both within and across health boards whilst also allowing for potential data saturation to be achieved [11]. The purposive decision was further shaped by available funding and time to conduct the study, ensuring that data saturation could be achieved within the practical constraints of the research.

### Data collection

Data were collected through semi-structured interviews conducted either individually or in small focus groups, depending on participant availability. Interviews were conducted via Microsoft Teams and audio-recorded using the platform’s built-in recording feature. Prior to each session, participants received a verbal briefing reiterating the study’s aims, confidentiality assurances, and their right to withdraw at any point. Informed consent, both verbal and written, was obtained for participation and for the use of anonymised quotations. Audio recordings were securely downloaded immediately following each session and stored on an encrypted research drive at the University of Stirling. Recordings were subsequently deleted from Microsoft Teams. Interviews were conducted between 27 April 2024 and 30 June 2024, with a mean interview duration of 38 minutes (range: 19–62 minutes). Transcripts were prepared verbatim for analysis.

Sample size was driven by principle of *data saturation*, which suggests that data collection can stop once no new insights emerge and the researcher begins to hear the same responses repeatedly. A purposive upper limit of 30 participants was,

### Data analysis

Main analysis was completed by two researchers (CS and DP) using discussion and reflection to achieve a rich interpretation of meaning [12].Then feedback from the wider project team was incorporated. An inductive thematic analysis was conducted using Microsoft Office (word and excel) software. Dependability of the research was achieved through an iterative process of checking author analysis against the transcribed data at different stages. The initial stage involved iterative reading of the transcripts to identify preliminary codes, generated through recurring words, phrases, and key concepts highlighted by the authors. Codes were reviewed and refined to ensure consistency; for example, similar terms such as “fast,” “quick,” and “speed” were consolidated under a single code, “speed.” Following initial coding, related codes were grouped into broader categories. For instance, “nuisance,” “reliability,” “noise,” and “invasion of privacy” were subsumed under the overarching code “The Drone,” with the individual elements treated as sub-codes. Coding was performed manually and iteratively, with ongoing cross-referencing of transcripts to ensure rigour. An inductive approach was adopted, which allows for the themes to be directly linked and driven by the data. Furthermore, a latent approach to the themes generated was undertaken. This allowed the researcher to identify or examine the underlying ideas and assumptions [12]. Subsequently, coded data were clustered into eight descriptive themes aligned with the study’s research questions. To enhance the credibility of the findings, the coding and thematic categorisation were collaboratively reviewed by two authors (CS, DP) through face-to-face meetings to resolve discrepancies and reach consensus. Credibility of the analysis was enhanced by the use of direct quotations to illustrate key findings and maintain transparency of the data.

## Results

### Participant demographics

A total of 26 participants were recruited across five Scottish health boards [10].Three participants were recruited who had an association with two health boards. That is they lived within one health board and worked for another. The cohort included nine female participants. Participants represented a diverse range of ages, backgrounds and healthcare experiences. That is, participants had experience either receiving healthcare as a patient, or were directly involved in the delivery of medical supplies and medications, specifically chemotherapy, laboratory diagnostics, and blood products. This study focused on perceptions of potential, rather than current, services, incorporating participant reflections on both personal experiences and anticipated future impacts (Table 1).

**Table 1:** Patient demographics.

Region	Number of participants	Age (mean ± /SD)	Sex (M/F)
Grampian	12	49.73 ± 15.91	M:9 F:3
Ayrshire & Arran	4	53.50 ± 19.16	M:3 F:0
Highland	7	54.29 ± 15.71	M:1 F:6
Other	3	36.00 ± 4.55	M:3 F:0

### Thematic analysis

Eight key themes emerged from the data analysis: (1) Acceptance, (2) Knowledge of drones, (3) Potential use within the NHS, (4) Potential impact, (5) Concerns, (6) Public perception, (7) Support network, and (8) Future innovation. A thematic map summarising codes and sub-codes alongside illustrative quotations are available in Table 2.

**Table 2:** Overview of themes, codes and sub-codes, and representative quotes.

Theme	Code	Sub codes	Contextual sub codes	Quotes
**Acceptance**	Negative	People not machines		*I really don’t see them coming in with respect to effectiveness. I think the effectiveness of, let’s call it good quality of healthcare really depends on the people who are providing the healthcare and not the machines.*
		Solve other problems		*It’s a distraction.*
	Positive	Evolving		*Absolutely. At one time you would never have done a GP consultation over facetime or whatever, you would have had to gone and travelled how many miles to see your GP. So, I think we must look at it positively.*
		Environment		*We rely too much on road transport, it’s always been roads, roads, roads, and there has to be something alternative.*
		Rural communities		*Yes, and for rural communities that are quite a distance away, and distance in time…so that’s a thing to consider, is distance in time rather than actual miles driven, it’s the time that is often…time interventions for drugs or equipment, that’s where I see a drone really helping rural communities.*
	Uncertainty	Not an easy process		*But I’m sceptical, I think is the bottom line. I mean, yes, it would be nice that you just launch a drone off the top of the pharmacy’s roof and off it goes. It’s very much not that simple.*
		Systems not ready		*I suppose, like other people, a bit mixed. I mean, on the one hand I think it’s potentially exciting, but I’m not fully convinced that the technology is at this stage now.*
		People not machines		*As I say, it depends on the local facilities as to what they’re actually doing, because if that person is needing an IV treatment, well no drone system or even van delivery system is going to help with that, they’re going to have to go to hospital.*
**Knowledge of drones**	Awareness	Media		*Well, they’ve been sort of in your face the last few years haven’t they, you see lots of media coverage on drones.*
		Operator		*Have been working within the drone environment. We are a company who provide drone solutions ideas to any number of people, one of which has been NHS Grampian.*
		General awareness		*You know, or some people have had the experience of an Amazon delivery using a drone or take away pizza arriving with a, you know, a mini drone.*
	Job roles	Surveillance/ Survey		*We get surveillance drones up there, obviously surveying the land quite a bit.*
		Delivery		*Seeing that drones are being used a lot more in the United States now, Amazon seem to be looking at now using them for delivering parcels.*
		Emergency services		*We obviously had the monkey that escaped from the Highland Wildlife Park, and it was just phenomenal when you’ve seen what they were able to locate, this tiny little monkey in such dense forest, that was amazing. Obviously with having the Cairngorms close by we’re seeing people who go missing, so we’re seeing where drones are being used by search and rescue teams to be accessing areas that maybe aren’t accessible by person.*
		Filming/ Photography		*I mean they’re used everywhere; I mean photographers swear by it, the BBC does a lot of drone coverage for the news.*
		Military		*Obviously, we know all about the military usage of drones.*
		Health care		*I’ve seen a few other places down south using drones, for medical purposes, in Northumberland for example, they use drones.*
		General commercial capacity		*A few commercial companies use them in most big cities. Also, I’ve seen a couple of them in agriculture where people around here use them on their farms and use them for agricultural processes.*
	Operational capabilities	General flying capabilities		*I am a licensed drone operator, not for any of the big ones, but just the small ones. But we have a couple of DJI’s, and so the whole principal of them, quite aware of it.*
		Regulation		*I know there are so many laws on where you can and can’t fly them.*
**Potential use within the NHS**	Emergency only			*I would think that it would be an extra service, and it wouldn’t be taking, well it ought not be taking from. Drones should be used to pick up the near misses or the fatalities or the critical incidents that our current coverage doesn’t catch.*
	Routine use			*But in the immediate, then it would be non-life-threatening things or non-immediate things.*
	Relevant tool for relevant task			*So, I feel it’s more about its crucial need and it being efficiently used, irrespective of area, irrespective of location.*
	Complimentary to current service			*Drones would be part of the system, wouldn’t be a complete replacement.*
**Potential impact**	Patient quality of service	Quality of Care		*I think to a larger extent there will be a contribution to quality of care. Although it could just be a catalyst towards quality of care, because it’s going to speed up the whole care process, because it’s a faster mobility aid, it’s a faster mode of transport of needed equipment or organs or samples to the sites where it is to be used. So, I think it’s going to have an impact on the quality of care.*
		Quality of Life		*I suppose the cost involved of transporting someone down to the mainland from an island for, say, a cancer consultation, if they can do that electronically, and medication can then be sent up to that person…[…]…so it might actually save people being transferred away from the local area. Because we know that people who are in a hospital environment, having friends and family around them is very beneficial for them, but if you have people being brought down from the islands they’re away from their family and friends, they’re very isolated, not able to access their own clothes, food, things like that as well. They’re paying excessive amounts of money for things, they’re having to rely on strangers to go and get them things, so people being in their own local community.*
	Leavers for change	Reach		*I don’t think anyone in a city would ever consider the need to have medicine transported by drone, because you’re within touching distance of anything, a blue light will get whatever is required. Whereas in rural areas, in the whole of the Highlands, and look at Argyll and Bute as well, it’s the time, it’s the time as well as the distance, it’s the time to get things delivered from your central hub to the local GP surgery.*
		Reliable service		*Because of the islands and the weather conditions, often you’ll see that the boats cannot come with the supplies…[…]...They were waiting two weeks for a delivery of drugs, specifically that they have to dispense to patients, so then it creates backlogs and bottlenecks in the whole system where you don’t have the reliability.*
		Building capacity		*I think it’s really important that they embrace and use technologies to the best effect as well. I think it could definitely build capacity as well, again, if the technology allows.*
		Mechanical reliability		*Yes, and it’s reliable, the drones are very reliable because they’re kept quite light, quite lightweight, so they’d be quite reliable, especially the electric ones I’ve seen.*
		Reducing errors		*Know there was an issue with samples that had got accidentally dropped at the side of the road and it was an accident…[…]…you get people that are tired and doing long hours and everything else, they make errors. We’re all human. You’re not going to have that problem with a drone if you’ve put tests into a drone to be carried from point A to point B. Your drone’s not getting tired. It’s got its flight path, and it goes for A to B and that.*
		Safety		*You get a guy driving an ambulance, that’s relatively speaking a risk, going from A to B, he’s charging down the road with his blue lights on. Whereas with a drone, it could well be you’re up in the sky, you haven’t got time, so you’re going to do it a lot quicker, and you’re going to do it a lot safer.*
		Speed		*When you are shifting materials between sites, again, it could be much easier to do so, simply because, again, you are not having to look at the traffic….[…]…there is so much roadworks now that it can be a bit of a nightmare. So even just saving those five minutes, it could potentially be saving a life at the end of the day.*
		Efficiency		*Use it for more things, that aren’t necessarily ad hoc? And then of course, when you do that, you come to the situation as to how Royal Mail, for example, or Amazon deliveries, or DPD, or all these guys, how they drive their efficiency, in the sense that they are doing the same routes every day. And that’s where the efficiency comes in, because you can cost out exactly what you’re doing, timewise and everything else. So, it would become more efficient to use it the more you do use it.*
	Additional benefits	Workforce development		*I think also there has a potential that with drones we might actually be able to see a change in people who are actually applying to work in more rural and remote areas, because they maybe will feel they’re maybe being more supported. It may actually have that ability to allow people to move to areas they want to live in.*
		Workforce generation		*I just thought if you if you if you were in a situation where in order to have drones it meant that you had to bring extra skilled people in. I might just be worth thinking about. Could those people be used for other things, other beneficial things as well? So that you have somebody there who say a skilled engineer. Maybe the drone wouldn’t. It wouldn’t fill all the time. Maybe with the right individuals and you know the right planning and so on, if you if you were bringing people in for this particular purpose. Maybe they could have some spin off benefits elsewhere.*
		Leading innovation		*Could be really beneficial for the UK as a whole, and Scotland, as being the innovators who started all this as well. So I could see it actually being something that could actually generate income for you in the future if you were to take it to other countries, other continents.*
		Environment		*Yes, so on the axis you were describing in terms of the time/efficiency/cost, I also think it’s worth including environment, because I know there is a governmental and society ambition to decarbonise a lot of the industries.*
		Cost		*If you start drawing comparisons. If you look at how things are done now. So, if you take the helicopter route, for example, £8,000 an hour, or whatever it cost, whereas a drone could be absolutely a very small percentage of that. You can control it from anywhere. It’s much less costly in terms of maintenance. You get a helicopter go down, in terms of maintenance time, that’s a long time. Whereas with a drone, much, much simpler. The whole infrastructure for a drone is a lot simpler than perhaps the way some things are done now.*
**Concerns**	The drone	Drone capability		*Maybe carrying larger equipment, might not be possible or feasible because of its size. I don’t know how the drones will be designed and what capacity it could carry, but the few drones I have come across are usually quite small and might not have the capacity to carry bigger equipment.*
		Nuisance		*Drones will be yet another human made item within the landscape. Drones, in a futuristic world, whatever our futuristic world is [laughs], there are just these drones’ whizzing past in the sky, and that I wouldn’t want. It should be an exception to the rule.*
		Reliability		*Reliability would be the key obviously for me, if it was a reliable service, and didn’t crash, and didn’t deliver to the wrong address on a regular basis, then I think it’s an advantage, I would be very much for it.*
		Noise		*Might be a negative issue if people found that they were unacceptably bothered by the noise of drones.*
		Invasion of privacy		*Drones usually carry cameras to detect where it’s going to, so some people might have this awkward feeling that it’s definitely…they might not have the understanding that it is for health purposes, and they might think it’s maybe a surveillance drone. So, I’m really thinking that might be a concern, because if it’s not carefully labelled people might not really understand the purpose of the drone. And even if it’s labelled it might still…what’s the guarantee that it’s not taking aerial photographs of places, of crowded places, and some scientific sites and sensitive areas.*
		Safety of device		*You’ve got cybersecurity. Because virtually everything can be hacked nowadays.*
		Safety of payload	Sample quality	*I suppose, again, it would be for the clinical side of staff, but if blood was taken up to a certain altitude or a certain height does it have an impact on the sample?*
		Safety of payload		*Could it go into the wrong hands. But that just has to be looked into, and obviously probability looked at, so I think we can always get round that sort of thing, whether it be something’s locked, something password protected.*
		Information governance		*A concern for me as well that obviously you’ve got. If you see it with samples, you’ve got the patient’s CHI number. Presumably the patient’s name, their address attached to that. You know, those are major information. You know the data breach concern would be what if somebody got into.*
		Public safety		*I mean also I mean if it came down, would there be a risk of damage to somebody’s property’s property and they would have to be insured properly in case that happened, I would assume. You know, or the risk of it hitting somebody if it came down.*
	The concept	Cost	Health boards	*Not all health boards are equal. Would there be some sort of cost implication here for the health boards? Would the less poor health boards be able to afford the drone service vs a very poor health boards deep in debt? Might not be able to afford it. Or would it be imposed nationally from above?*
			Appropriate services	*The counter argument, or the counter thought, could be, surely, they should be spending money on putting more doctors in the doctors’ surgeries, or something as basic as that. And you can understand that. And that’s not necessarily maybe an objection to the concept or the process of using drones to do this, it’s more a concept of, I’d rather it was just easier to see a doctor.*
			Patients	*In terms of the cost, like I said, I’m thinking we have to involve drones and there might be some kind of extra charges or maybe they might say, if you want samples to be transported within a day, then you pay this.*
		Political		*Things like this kind of idea and it could easily very easily go political and Scotland’s very political at the minute.*
	Impact on current process	Jobs and staff	Jobs neutral, Jobs positive, Jobs negative	*There is also the social aspect, where I’ve spoken to people who will say to you ‘I quite like the local boy Michael, who’s always driven around and dropped these things off, popping around, and I don’t want Michael to be replaced by something like that’, so I think that also needs to be considered sensitively.*
			Recruiting staff	*Because the difficulties getting the staff, you know where we are. This is not just something that affects the health service. It’s the same with teachers and you know, I know of a bus driver who’s been living in his car. He couldn’t find anywhere to rent and. Because, you know, I live in the National Park. And people, it’s a place that people want to come to, for Airbnb and second homes and you know, holiday lets and so on. There is great difficulty in you know, even when people want to come and live here.*
		Adoption	Embedding into system	*The NHS, is that the current use cases, they’re sort of embedded in a system that’s been going for 50 years or more, and to have something new come along like a drone is something that’s going to be quite a hard sell.*
			Clinical staff burden	*I’m looking at what form of training? First off, what form of training will be given to doctors? Or is it doctors that are going to handle the drones or are they going to be handled by a special sector that will be created within the hospitals that we ensure that this service is functional and working?*
			Service burden	*Depends on the frequency. If it’s only once a day it’s coming in, it’s not really a big thing for somebody just to go out and get a notification that it’s coming at whatever time, and go out and take a package, and pick up another package, for the return flight. That’s not a big deal. But if it’s happening six times a day, seven times a day, then that’s probably a bigger drain on their resources, that budget wise, they’re probably just not going to have.*
			Not wanting change	*Staff can be. Quite kind of, well, we have this system, and the systems works, and we don’t want to change it….[…]…people in general are unwilling to change. That’s human nature. They’ve got their safe zone and they’re safe in this and that this works and that’s fine.*
			What is the alternative	*I can see you’ve got a problem as you would with road transport as well. If you’ve got an absolutely critical sample, that really must get through and then either the road is blocked, or the weather is not right. Flying or there is some kind of rare but catastrophic event like a wildlife thing or technical failure. Then you know I could see there would be the challenge of the sample or the critical piece of equipment or whatever it might be hasn’t arrived. That could be life threatening in the in the in the extreme case, it’s one of those. High hazard, low risk Situations.*
		Interoperability/ siloed care		*Will there be one drone service? Cause the NHS trusts are notorious for going their own way and having incompatible units, as in software and machinery, etcetera. Would there be one drone service? And drones, would they be? Interchangeable. Because the border between health districts is fairly small. So, if something’s on the border, would it make sense to fly out with that one particular trust and go and get serviced and reloaded at another trust.*
	Implementing the service	Operational planning	Connectivity networks	*You need 100% and instant communication between the drone pilot and the receiver. That would have to be instantaneous, and it would need to be continuous, be it a mobile phone or whatever. Remember, a lot of these areas, you will not get mobile phone coverage.*
			Flight planning	*Highlands are used by the RAF, as I mean there’s a bombing range and so forth. So, you’ve obviously got that to contend with. So, I can’t work out in my head how you would make that work, that would be a concern for me, as is what would happen around the planes that are already in the air and the things that are already up there.*
			Mitigating risk	*So, it might have accidents, it might have unforeseen circumstances, and the drone definitely might not reach its predestined destination, but all of these are just a handful of the factors that need to be considered in this innovation and what could really be done to mitigate and reduce these hazards while flying drones.*
			Accountability of process	*I would be less concerned if someone is eventually in the drone and controlling it. But if someone is in the airport or whatever, centre, and it’s just remotely controlled, I might be more concerned because I don’t know how well the job could be done. But if someone was inside, they would also know that their safety is involved, so they have to be very careful. But then if someone is just sitting in the office and making controls or moving a joystick, they might not really be concerned because they are not personally implicated in the problem.*
		Regulations	CCA	*Beyond visual line of sight was top of the pile then, and here we are now, and we’ve moved it forward, but in my view not sufficiently to keep up with the technology. The technology is there, but this infrastructure is not keeping pace with it..[...]...And the fact that drones is developing, we can take a backseat there, and just maybe we’ll address it in a year or two’s time. My concern would be that we’re going to get left behind.*
			Operator skill	*Training of operators, they should be able to direct drones from both sides, from the bigger hospitals and the smaller hospitals, they should be able to train the operators*
		Environment	Geography and weather	*Well, your small drones are only five metres per second, which isn’t that windy. But the bigger they go, they are far more capable of dealing with big winds and so forth. But we do get some fairly decent storms up here over the winter months. Even to the point that the helicopters that are flying off to the rigs just simply don’t go. So, it does happen.*
			Scenery	*However, my concern would be that, like the passage of pylons around our very lovely empty landscapes, that we’d start to have really busy flight lines.*
			Wildlife	*Wildlife could be an issue. I mean not only a question of could you get to you know birds you know I don’t think bird strikes as much as birds attacking a drone, but because of where we are with the high conservation focus on this area. It’s an interesting question where you would be if it was, you know, a case of Golden Eagle or Osprey versus drone. You know, where would the priorities be seen? You know, around here people put a very high value on Ospreys and Golden Eagles.*
**Public perception**	Implementation	Local benefits		*I think some of these rural areas, they’re generally a very accepting community, because they just don’t have that infrastructure. So I think we might see that first, being deployed and trialled, and then slowly reaching out and spanning out further.*
		Service benefits		*In terms of NHS, I think people read stories now about, you see the NHS are using drones to do that to that, that sounds wonderful, in theory, because they can see benefits to that, as long as it doesn’t affect them too much.*
		Change to current systems		*In terms of the public, the public’s view of drones is a little bit, not in my back yard, a little bit, I’m not sure about them, because I don’t know enough about them, a little bit, Are they noisy? A little bit, are they going to interfere with my leisure? I mean, we’ve flown in places, for example, the local aero modellers club, they get up in arms about the fact that we want to take some of their space for a period of time. So, it’s a little bit, well, there’s change, and do I want to accept that change, because it’s not my problem now?*
		Cost		*At the beginning, people are going to see it as it’s going to cost more money for the NHS.*
		Wider NHS issues		*Well, it depends how much money you’re going to devote to a drone programme. Is it going to take money away from other things? That’s what you’ve got to look at. That’s what people look at, is if you’re going to take one of the doctors out of their pharmacy so you can finance a drone project. Yeah, I wouldn’t be happy about that. But, again, if I lived out in the sticks and I didn’t have a doctor within 50 miles or 100 miles.*
		Political concept		*They worry about the politics of it, and they think, Mm, is this something we want to push right now? Because, I wouldn’t say it’s a bit sticky, but it’s not a sure thing. There are other things they’ve got, I think, on their agenda, where perhaps it’s more pressing, or it’s more directly translatable into the needs of us, the taxpayer.*
	Drones	Technologically prepared		*I think you might have some fairly large clues. From elsewhere outside your project, because I won’t be at all surprised if five years from now or 10 years from now. People aren’t, at least occasionally, you know, or some people have had the experience of an Amazon delivery using a drone or take away pizza arriving with a, you know, a mini drone.*
		Nuisance		*Yes, I think the nuisance problem, I’ve been online and gone into a few chat rooms and things, where down in London or down south there’s lots of drones all the time, and people are just finding them a nuisance.*
		Lack of knowledge		*I don’t think we can really assess that. I don’t think we are informed enough yet because I’m definitely sure there are other questions that will be raised. So, if I were to tell someone about this before now, I wouldn’t really know what to say, I would just say. They want to use drones. I don’t know how the drones look, I don’t know what they are going to do, whether they carry people or not.*
		Safety		*Yeah, I think I think there the general public worry about things getting lost or things. You know what would happen if it broke down?*
		Ulterior motive		*People worried about their own security, their own houses, and what the drone is doing in the first place. People are very wary of new technology sometimes, especially when it’s used against them. Now I know the police have got a couple of drones they use down south, checking traffic for speed. So where there’s a positive to this, there’s also a negative, where people are wary of the drones, what are they really there for, are they spying on us, what do they want, is it all going back to Big Brother’s watching us all over again.*
**Support network**	Ownership	Control	NHS Control	*I’d want the thing totally controlled by the NHS. Having had experience of, let’s say, systems. I would only want the NHS to be in control of it.*
			Partnering with industry	*I think the realistic side of that is that it would have to be tendered, I would think, simply because nothing like this is in the NHS’s wheelhouse. I think there’s probably numerous cases where they’ve tried to do something in-house, and it’s just a disaster, because they don’t have the expertise to deal with it.*
		Partnering with other services		*I’m initially thinking where something’s going to land potentially, but then actually maybe it can land at a police station or a fire station, and they’re equipped to do that, and then from them it’s only a two-minute drive for them to take it to the local hospital.*
	Clinical	Clinical staff		*There’s amazing staff already on the Islands. So, say, if the drug were already shipped to the patient or to the hospital to then be transported to the patient, it could simply be a trained nurse. An ANP, for example, could go along and it is literally just connecting it up to your central line or your pick line, depending on which type it is you’ve got. And it can even be taught to some patients.*
		Patient choice		*It’s putting it back onto the patient. Do you want to do this at home, or would you prefer to be in hospital? And it is patient-centred, focused care, and that’s what we are all aiming for right now*
		Operational planning (clinical)		*So, whether that’s to get replenishment of tablets, because they have a short life date, or whether it’s for other things, I don’t know. But obviously people who are on that sort of treatment need to be monitored very, very carefully for just even basic things like temperature, to highlight any potential signs of infection and so forth.*
	Flight support	Flight infrastructure		*Absolutely it could. That’s exactly the sort of thing. But again, I come back to the point that a helipad at a hospital needs that big surrounding area to make things safe….[…]…Whereas with a drone, it just comes out of the sky, and you could do it on a very small area. So, there is a big difference.*
		Flight workforce	Maintenance	*At least a minimum of ground crew at both ends. Because if it arrives with the technical fault and it’s saying Caithness, you really don’t want to have to be sitting a drone onto a lorry and shipping it back to its start place you know, a maintenance support team in Inverness or if it’s been flown from even further afield.*
		Regulation		*Some laws challenging airspace movement should be really addressed to make sure that this area could accept policies that accept flying within a particular jurisdiction.*
		Sustainability		*I would draw the parallel to an issue which we have around here sometimes, people will create a footpath and they’ll find the money to create a footpath. But they don’t provide the resources to maintain it. And you know with any project like this, it’s very important that you don’t just find the money to set it up. You also guarantee its future.*
		Operational planning	Environment	*I mean, any time you have a flight, you need to produce an operational safety case. You have to consider quite a few elements, of which the environment and nature plays a big part. And you get stakeholder discussions and agreements; these sorts of things are usually raised at that level. If you’ve got a flight path for birds, that’s dialled into your operational safety case, you are aware of it before you fly.*
		Operational planning	Efficiency	*I would think, whether you’re doing single drops and returns…[…]…is it taking off from Aberdeen with a 30-kilogram payload? Is it only going to one location, or is it going to Fraserburgh, and then it’s going to Peterhead, and then it’s going to return to Aberdeen? Because you’ve got an efficiency argument there as to which is more efficient. It depends on how much stuff is going, and how time critical, for example, it would be prudent to then put something that needs to go from Fraserburgh back to Aberdeen in the return flight. And obviously then there needs to be the back-end systems that work out what the weight of that is, and is it acceptable to send it...[...]...I think obviously something on this is being looked at to improve efficiency, and to improve delivery of things, so looking at all aspects of that is really important. And say, to fly effectively past one hospital, or one rural location, to go to another, that may have to happen from time to time.*
	Public support	Support network - communication		*I think the message for me is always that there are huge advantages to one system over another system, and that I think it’s the things that people view as important, and I think speed, quite often with a medical situation, with a medical emergency, is one thing people would appreciate. If you can say, that’s going to get there by drone in one hour, as opposed to four hours on the road, I know which they would choose every time.*
**Future innovation**	Emergency care and treatment	Emergency treatment		*Well, the emergency side is obvious because it gives you basically instant response because you could have drones that have got analysing equipment on them. So, you could basically strap the drone on somebody’s arm doing an analysis there. Radio it back to base, you’ve got an instant answer. Yeah, for your emergency thing, there was a bit there that shows putting a defib on a drone. Yeah, that’s a bit dramatic. But, yeah, if somebody’s stuck somewhere and you need a defib in a hurry, that could save a life.*
		Communication device		*So, if a doctor can see what’s going on with a patient while a crew’s trying to get there. Even a case of if somebody’s passed out, even somebody speaking to them over a drone, just to try and keep them alert, trying to keep them speaking has that potential for them to be able to actually assess the GCS and things like that. So, there’s that potential as well. I’m probably going way too far.*
	Additional services	GP deliveries		*Maybe you will first see the local GP practice having those deliveries enabled by that, before you get door to door drop offs.*
		Localised delivery		*I know this technology would not be quite easy, but from clinics, or even from pharmacies, there should be some sort of delivery. And it should be used also more commercialised…[…]…A lot of people should be able to order and receive medications even at the comfort of their homes using drones. And it could be way faster, and that improves the quality of care generally.*
		Moving people		*If you’ve got patients that need moving from A to B, and you’ve got patient confidentiality, you’ve got things like Covid, maybe, it might be that the use of an appropriate drone could be a better way of achieving that than patient transport, for example, or a helicopter.*
		Alternative drone methods		*We’re talking about drones being in the air. In the Western Isles, would there be a possibility of drones by boat. A little autonomous boat. You could certainly carry a heavier load. You could possibly go through rougher seas.*

### Acceptance

Most participants expressed enthusiasm regarding the potential adoption of drones in the NHS, highlighting perceived benefits such as environmental improvements and enhanced service delivery to rural and isolated communities.

*“Yes, and for rural communities that are quite a distance away, and distance in time…so that’s a thing to consider, is distance in time rather than actual miles driven, it’s the time that is often…time interventions for drugs or equipment, that’s where I see a drone really helping rural communities.”*

Drones were viewed as a necessary evolution of healthcare systems, offering an opportunity to innovate traditional logistics processes.

*“Absolutely. At one time you would never have done a GP consultation over telehealth or whatever, you would have had to gone and travelled how many miles to see your GP. So, I think we must look at it positively.”*

However, a minority voiced reservations, noting potential challenges in embedding new technology within existing systems.

*“I suppose, like other people, a bit mixed. I mean, on the one hand I think it’s potentially exciting, but I’m not fully convinced that the technology is at this stage to be rolled out now.”*

Some participants emphasised the irreplaceable human element in healthcare, suggesting that while drones might enhance logistics, the core of healthcare delivery remains dependent on human interaction. A small number expressed scepticism, prioritising investment in healthcare staff over technological interventions.

*“As I say, it depends on the local facilities as to what they’re actually doing, because if that person is needing an IV treatment, well no drone system or even van delivery system is going to help with that, they’re going to have to go to hospital.”*

### Knowledge of drones

Participants generally reported broad, though non-specific, knowledge of drones, informed by media exposure and personal or occupational experience. Recognised applications included surveillance, military operations, environmental mapping, commercial delivery, emergency services, and entertainment.

*“Seeing that drones are being used a lot more in the United States now, Amazon seem to be looking at now using them for delivering parcels.”*

Only a minority were aware of previous healthcare applications, such as the delivery of defibrillators in Sweden or blood products in Rwanda [5].

*“BBC radio podcast about drones being used in Sweden, and I’m sure that it was to take a defibrillator to somebody who needed their heart restarted…or rather, their heart stopped, and then restarted.”*

Few participants had detailed knowledge of drone capabilities in medical logistics, highlighting a gap in public understanding of technological maturity and regulatory frameworks.

*“Aircraft have to be certified, drones have to be certified, you need flight permission, I think those things, that’s the big talking point, I suppose, in the industry, where we’re trying to move things forward, but we can only go forward at a certain pace.”*

### Potential use within the NHS

Participants identified several potential applications for drones within healthcare. Emergency logistics, such as the transport of blood products, medications, or organs, were prioritised.

*“I would think that it would be an extra service, and it wouldn’t be taking, well it ought not be taking from other resources. Drones should be used to pick up the near misses or the fatalities or the critical incidents that our current coverage doesn’t catch.”*

Some participants envisioned broader routine use, although there was uncertainty regarding operational limitations, such as payload capacity, flight range, and service reliability. The majority recommended a complementary model, where drones enhance, rather than replace, existing transport services.

*“Drones would need to be part of the system, but it wouldn’t be a complete replacement.”*

### Potential impact

Although participants were cautious in predicting specific impacts due to limited knowledge of drone operational capabilities, several hypothesised benefits were proposed. Drones were perceived as potentially enhancing the quality and timeliness of service delivery, particularly in rural areas.

*“I think to a larger extent there will be a contribution to quality of care. Although it could just be a catalyst towards quality of care, because it’s going to speed up the whole care process, because it’s a faster mobility aid, it’s a faster mode of transport of needed equipment or organs or samples to the sites where it is to be used. So, I think it’s going to have an impact on the quality of care.”*

The ability to receive care closer to home was seen as beneficial for reducing travel burdens, mitigating associated financial costs, and improving psychosocial wellbeing.

*“I suppose the cost involved transporting someone down to the mainland from an island for, say, a cancer consultation, if they can do that electronically, and medication can then be sent up to that person…[…]…so it might actually save people being transferred away from the local area.”*

Participants also suggested potential system-level benefits, including reduced transportation errors and increased service reliability, particularly in adverse weather conditions or where conventional transport is limited.

*“Because of the islands and the weather conditions, often you’ll see that the boats cannot come with the supplies…[…]...They were waiting two weeks for a delivery of drugs, specifically that they have to dispense to patients, so then it creates backlogs and bottlenecks in the whole system where you don’t have the reliability.”*

Additional benefits cited included workforce development opportunities, local economic stimulation, and environmental advantages through reduced reliance on fossil fuels.

*“I just thought if you were in a situation where in order to have drones it meant that you had to bring extra skilled people in. I might just be worth thinking about. Could those people be used for other things, other beneficial things as well? So that you have somebody there who say is a skilled engineer. Maybe the drone wouldn’t. It wouldn’t fill all the time. Maybe with the right individuals and you know the right planning and so on, if you if you were bringing people in for this particular purpose. Maybe they could have some spin off benefits elsewhere.”*

### Concerns

While direct concerns were limited, participants identified several key areas requiring attention to ensure safe and effective integration of drones into healthcare services. Technical concerns included payload limitations, flight reliability, and noise pollution.

*“Maybe carrying larger equipment, might not be possible or feasible because of its size. I don’t know how the drones will be designed and what capacity it could carry, but the few drones I have come across are usually quite small and might not have the capacity to carry bigger equipment.”*

Privacy and data security issues were also raised, with participants expressing unease about potential surveillance capabilities. Safety concerns focused on the risk of cyber interference, physical tampering, and the potential for malfunctions impacting public safety.

*“A concern for me as well that obviously you’ve got. If you see it with samples, you’ve got the patient’s CHI number. Presumably the patient’s name, their address attached to that. You know, those are major information. You know the data breach concern would be what if somebody got into.”*

*“I mean also I mean if it came down, would there be a risk of damage to somebody’s property’s property and they would have to be insured properly in case that happened, I would assume. You know, or the risk of it hitting somebody if it came down.”*

Participants questioned how drones would be funded and managed, raising issues of financial equity between health boards and the risk of deepening existing health inequalities.

*“Not all health boards are equal. Would there be some sort of cost implication here for the health boards? Would the less poor health boards be able to afford the drone service vs a very poor health boards deep in debt? Might not be able to afford it. Or would it be imposed nationally from above?”*

*“The counter argument, or the counter thought, could be, surely, they should be spending money on putting more doctors in the doctors’ surgeries, or something as basic as that. And you can understand that. And that’s not necessarily maybe an objection to the concept or the process of using drones to do this, it’s more a concept of, I’d rather it was just easier to see a doctor.”*

Concerns were also voiced regarding regulatory oversight, environmental impacts, and the potential strain on clinical staff who might be required to assume new operational responsibilities.

*“Depends on the frequency. If it’s only once a day it’s coming in, it’s not really a big thing for somebody just to go out and get a notification that it’s coming at whatever time, and go out and take a package, and pick up another package, for the return flight. That’s not a big deal. But if it’s happening six times a day, seven times a day, then that’s probably a bigger drain on their resources.”*

Resistance to change and challenges in integrating drone services with existing NHS infrastructure and IT systems were highlighted as potential barriers.

*“Will there be one drone service? Cause the NHS trusts are notorious for going their own way and having incompatible units, as in software and machinery, etcetera. Would there be one drone service? And drones, would they be? Interchangeable. Because the border between health districts is fairly small. So, if something’s on the border, would it make sense to fly out with that one particular trust and go and get serviced and reloaded at another trust.”*

### Public perception

Participants expressed mixed views on how the wider public might respond to drone integration. While there was general optimism that the public is increasingly accustomed to technological solutions, some participants anticipated resistance due to perceptions of drones as intrusive or unnecessary.

*“In terms of NHS, I think people read stories now about, you see the NHS are using drones to do that to that, that sounds wonderful, in theory, because they can see benefits to that, as long as it doesn’t affect them too much.”*

*“Well, it depends how much money you’re going to devote to a drone programme. Is it going to take money away from other things? That’s what you’ve got to look at. That’s what people look at, is if you’re going to take one of the doctors out of their pharmacy so you can finance a drone project. Yeah, I wouldn’t be happy about that. But, again, if I lived out in the sticks and I didn’t have a doctor within 50 miles or 100 miles.”*

*“because I won’t be at all surprised if five years from now or 10 years from now. People aren’t, at least occasionally, you know, or some people have had the experience of an Amazon delivery using a drone or take away pizza arriving with a, you know, a mini drone.”*

Public acceptance was seen as contingent on clear communication, visible service benefits, and assurances of safety and reliability.

*“People worried about their own security, their own houses, and what the drone is doing in the first place. People are very wary of new technology sometimes, especially when it’s used against them. Now I know the police have got a couple of drones they use down south, checking traffic for speed. So where there’s a positive to this, there’s also a negative, where people are wary of the drones, what are they really there for, are they spying on us, what do they want, is it all going back to Big Brother’s watching us all over again.”*

### Support network

Participants identified robust support networks as critical to successful drone integration. Views differed on the optimal model for ownership and operation; some advocated for NHS-led services to ensure cost-effectiveness, while others supported partnerships with industry or emergency services to leverage existing infrastructure and expertise.

*“I think the realistic side of that is that it would have to be tendered, I would think, simply because nothing like this is in the NHS’s wheelhouse. I think there’s probably numerous cases where they’ve tried to do something in-house, and it’s just a disaster, because they don’t have the expertise to deal with it.”*

*“I’d want the thing totally controlled by the NHS. Having had experience of, let’s say, systems. I would only want the NHS to be in control of it.”*

Regardless of model, participants stressed the need for clear regulatory frameworks, operational planning, and workforce training.

*“At least a minimum of ground crew at both ends. Because if it arrives with the technical fault and it’s saying Caithness, you really don’t want to have to be sitting a drone onto a lorry and shipping it back to its start place you know, a maintenance support team in Inverness or if it’s been flown from even further afield.”*

*“I mean, any time you have a flight, you need to produce an operational safety case. You have to consider quite a few elements, of which the environment and nature plays a big part. And you get stakeholder discussions and agreements; these sorts of things are usually raised at that level. If you’ve got a flight path for birds, that’s dialled into your operational safety case, you are aware of it before you fly.”*

Flexibility to adapt to changing service demands and ongoing community engagement were deemed essential to building public trust and ensuring service sustainability.

*“I would draw the parallel to an issue which we have around here sometimes, people will create a footpath and they’ll find the money to create a footpath. But they don’t provide the resources to maintain it. And you know with any project like this, it’s very important that you don’t just find the money to set it up. You also guarantee its future.”*

### Future innovation

Participants demonstrated considerable optimism about the future potential of drone technology in healthcare. Suggestions extended beyond logistics to include emergency diagnostics, remote treatment delivery, and enhanced communication capabilities for first responders.

*“Well, the emergency side is obvious because it gives you basically instant response because you could have drones that have got analysing equipment on them. So, you could basically strap the drone on somebody’s arm doing an analysis there. Radio it back to base, you’ve got an instant answer. Yeah, for your emergency thing, there was a bit there that shows putting a defib on a drone. Yeah, that’s a bit dramatic. But, yeah, if somebody’s stuck somewhere and you need a defib in a hurry, that could save a life.”*

*“So, if a doctor can see what’s going on with a patient while a crew’s trying to get there. Even a case of if somebody’s passed out, even somebody speaking to them over a drone, just to try and keep them alert, trying to keep them speaking has that potential for them to be able to actually assess the GCS and things like that. So, there’s that potential as well. I’m probably going way too far.”*

Broader innovations, such as drone-enabled medication delivery to patients’ homes and even patient transport, were discussed, although participants acknowledged that such developments would require overcoming significant regulatory and operational hurdles.

*“I know this technology would not be quite easy, but from clinics, or even from pharmacies, there should be some sort of delivery. And it should be used also more commercialised…[…]…A lot of people should be able to order and receive medications even at the comfort of their homes using drones. And it could be way faster, and that improves the quality of care generally.”*

## Discussion

This study explored patient perceptions of drone integration into healthcare logistics, focusing on cost and efficiency, safety and reliability, and quality of life. Overall, participants demonstrated a general familiarity with drone capabilities and expressed broad acceptance of their potential use in the NHS. While acknowledging the complexity of implementation, participants saw drones as a promising innovation capable of enhancing healthcare delivery, particularly by reducing logistical barriers for patients in rural areas and contributing to broader societal benefits such as environmental sustainability. Participants response suggested that drones could enable care closer to home, potentially improving both the quality of care and patient quality of life by reducing travel burdens and associated costs. However, successful implementation would require careful attention to clinical, infrastructural, and regulatory challenges. Addressing public concerns and ensuring a sustainable, integrated approach were viewed as essential to gaining acceptance and realising the potential benefits.

### Cost and efficiency

Cost and financing emerged as critical factors influencing the perceived viability of drone services. Participants voiced concerns that drones could become a politically charged issue and questioned whether resources might be better allocated to more pressing healthcare needs, such as staffing. These views align with broader findings that local communities often feel disconnected from commissioning decisions when service changes are made without sufficient consultation. To avoid exacerbating health inequalities, there is a need for collaborative, equitable funding models. Participants were uncertain whether drones should be funded and managed at the local health board level or through national directives. Without a coordinated approach, smaller boards might struggle to sustain such services, exacerbating disparities. Lessons from international models, such as the heavily subsidised drone programs in Ghana, suggest that centralised support may be an important lever for long-term sustainability [5,13].While the cost-effectiveness of drones remains uncertain, pilot studies have indicated consideration for operational costs relative to standard logistics [14,15]. Although short-term savings have often been prioritised within the NHS (12), this emphasis can sometimes reduce scope for longer-term transformational progress. A shift in focus from narrow cost calculations to broader assessments of holistic value-incorporating patient outcomes, access equity, and environmental impact-is likely essential for justifying drone integration.

### Safety and reliability

Despite significant technological advancements, drones remain an emerging mode of medical transport with limited long-term safety data. Public perceptions of risk are influenced by personal knowledge and perceived benefit, highlighting the importance of clear communication and transparency during implementation. Participants identified drones’ potential to mitigate some challenges faced by road transport, such as delays due to traffic or road closures. However, weather was highlighted as a critical factor, particularly in rural and remote areas where wind and storms could impact drone operations more significantly than ground transport [16]. While drone designs vary in resilience to weather conditions, the variability across the UK-particularly between the south and northern Scotland-necessitates robust contingency planning. Current evidence on drone reliability and specimen integrity is encouraging, showing minimal impacts on the stability of transported medicines such as insulin and the accuracy of laboratory specimens [17,18]. Nonetheless, further research is needed to assess performance over longer distances, varying weather conditions, and with different payloads.

### Quality of life

Decentralisation of healthcare services is associated with improved patient satisfaction and reduced logistical burdens, although achieving equitable access remains a challenge [19]. Participants in this study emphasised that while drones may not directly impact clinical decision-making or treatment quality, they could significantly reduce travel time, costs, and stress, especially for patients in remote areas. Social support has been shown to correlate with better health outcomes and reduced hospitalisation rates [20], while social isolation negatively impacts psychological wellbeing and care experiences [21]. By facilitating more local access to healthcare services, drones could contribute to reducing isolation and enhancing patients’ social connectedness during treatment. While initial studies on drone transport of medical products are promising [20,22], additional research is required to validate long-term efficacy and address preanalytical challenges associated with specimen transport. Practical innovations such as volunteer Blood Bikes services in Scotland demonstrate the benefits of expanded logistics capacity, and integrating drones could build upon such models [23].

### Distinctions by payload type

Although the study materials prompted discussion across chemotherapy, blood products and laboratory diagnostics, participants did not consistently distinguish between these payload types in their views. Instead, perceptions were largely driven by *how* drone-enabled logistics would be implemented-particularly safety, reliability, governance, privacy and equitable access-rather than *what* was being transported. This is an important finding for implementation: building trust may depend less on the specific clinical item and more on clear communication about operating models, risk management, chain-of-custody, and accountability. Future work could use condition-specific sampling (e.g., people currently receiving chemotherapy) to explore whether item sensitivity shapes acceptability when participants have direct lived experience of the relevant pathway.

## Limitations

This study offers valuable insights into patient perspectives on drone integration within medical logistics, but several limitations should be acknowledged. First, the findings reflect views from a relatively small sample of participants across five Scottish health boards and may not be generalisable to other

populations or regions. Second, the study focused exclusively on patient perceptions and did not capture the views of healthcare professionals, logistics providers, or policymakers. Third, while participants discussed hypothetical and anticipated impacts, many had limited direct experience with drones, which may have influenced the depth or specificity of responses. Finally, the study did not explore detailed technical or operational dimensions such as cost modelling, regulatory frameworks, or real-world performance metrics, which would require complementary research approaches.

## Recommendations and implications

Findings from this study highlight the importance of a cautious and context-sensitive approach to drone integration. Key considerations for implementation include:

- Defining the intended role of drones (specialised versus generalist applications).
- Establishing clear success criteria and evaluation frameworks.
- Ensuring integration with existing care pathways to minimise service disruption.
- Developing robust support networks encompassing clinical, technical, and public stakeholders.
- Prioritising co-design with patients, clinicians, and local communities to foster acceptance and ensure services meet user needs.
- Operational factors such as interoperability, workflow alignment, chain-of-custody, and cold-chain logistics should also be considered in future planning and evaluation.

## Conclusion

This qualitative study provides important insights into patient perceptions of drone integration within medical logistics. Across five Scottish health boards, participants expressed general acceptance of the technology and optimism about its potential to improve healthcare access, efficiency, and quality of life, particularly for those living in rural or underserved communities. However, successful implementation depends on addressing practical concerns around safety, regulation, cost, interoperability, and public trust. Drone technology alone will not resolve systemic challenges in healthcare logistics, but it may serve as a valuable complementary tool within a broader strategy for service innovation. To fully realise its potential, healthcare systems must continue to invest in evidence-based implementation, co-design with communities, and integrated planning across technical, clinical, and policy domains. Ultimately, drones could contribute to a more sustainable, equitable, and patient-centred model of care if developed and deployed with foresight, rigour, and meaningful stakeholder engagement.
